# Multi-spectral immunofluorescence evaluation of the myeloid, T cell, and natural killer cell tumor immune microenvironment in chordoma may guide immunotherapeutic strategies

**DOI:** 10.3389/fonc.2022.1012058

**Published:** 2022-10-21

**Authors:** Diana C. Lopez, Yvette L. Robbins, Joshua T. Kowalczyk, Wiem Lassoued, James L. Gulley, Markku M. Miettinen, Gary L. Gallia, Clint T. Allen, James W. Hodge, Nyall R. London

**Affiliations:** ^1^ Sinonasal and Skull Base Tumor Program, National Institute on Deafness and Other Communication Disorders, National Institutes of Health, Bethesda, MD, United States; ^2^ Cleveland Clinic Lerner College of Medicine, Cleveland Clinic, Cleveland, OH, United States; ^3^ Section on Translational Tumor Immunology, National Institute on Deafness and Other Communication Disorders, National Institutes of Health, Bethesda, MD, United States; ^4^ Center for Immuno-Oncology, National Cancer Institute, Center for Cancer Research, National Institutes of Health (CCR, NIH), Bethesda, MD, United States; ^5^ Laboratory for Pathology, Center for Cancer Research, National Cancer Institute, Bethesda, MD, United States; ^6^ Department of Otolaryngology-Head and Neck Surgery, Johns Hopkins University School of Medicine, Baltimore, MD, United States; ^7^ Department of Neurosurgery, Johns Hopkins University School of Medicine, Baltimore, MD, United States

**Keywords:** chordoma, tumor immune microenvironment (TIME), immunotherapy, myeloid cells, T cells, natural killer cells

## Abstract

**Background:**

Chordoma is a rare, invasive, and devastating bone malignancy of residual notochord tissue that arises at the skull base, sacrum, or spine. In order to maximize immunotherapeutic approaches as a potential treatment strategy in chordoma it is important to fully characterize the tumor immune microenvironment (TIME). Multispectral immunofluorescence (MIF) allows for comprehensive evaluation of tumor compartments, molecular co-expression, and immune cell spatial relationships. Here we implement MIF to define the myeloid, T cell, and natural killer (NK) cell compartments in an effort to guide rational design of immunotherapeutic strategies for chordoma.

**Methods:**

Chordoma tumor tissue from 57 patients was evaluated using MIF. Three panels were validated to assess myeloid cell, T cell, and NK cell populations. Slides were stained using an automated system and HALO software objective analysis was utilized for quantitative immune cell density and spatial comparisons between tumor and stroma compartments.

**Results:**

Chordoma TIME analysis revealed macrophage infiltration of the tumor parenchyma at a significantly higher density than stroma. In contrast, helper T cells, cytotoxic T cells, and T regulatory cells were significantly more abundant in stroma versus tumor. T cell compartment infiltration more commonly demonstrated a tumor parenchymal exclusion pattern, most markedly among cytotoxic T cells. NK cells were sparsely found within the chordoma TIME and few were in an activated state. No immune composition differences were seen in chordomas originating from diverse anatomic sites or between those resected at primary versus advanced disease stage.

**Conclusion:**

This is the first comprehensive evaluation of the chordoma TIME including myeloid, T cell, and NK cell appraisal using MIF. Our findings demonstrate that myeloid cells significantly infiltrate chordoma tumor parenchyma while T cells tend to be tumor parenchymal excluded with high stromal infiltration. On average, myeloid cells are found nearer to target tumor cells than T cells, potentially resulting in restriction of T effector cell function. This study suggests that future immunotherapy combinations for chordoma should be aimed at decreasing myeloid cell suppressive function while enhancing cytotoxic T cell and NK cell killing.

## Introduction

Chordoma is a rare, invasive, and devastating bone malignancy (1, 2). Chordomas arise from residual notochord tissue within the axial skeleton (1–3), and are therefore found in the clivus of the skull base, spine, or sacrum (1, 2). Prognosis is poor with reported five and ten-year survival rates of approximately 70% and 40%, respectively (3, 4). When feasible, standard treatment typically consists of surgical resection and radiotherapy. However, the tumor’s invasive nature and close proximity to critical neurovascular structures makes a negative margin or gross total resection difficult (2, 3). Furthermore, chordoma’s resistance to traditional chemotherapy and radiation is well established (5), and primary and adjuvant treatment strategies come with significant morbidity (1, 6). Thus, there is an unmet need for additional or targeted treatment options, and the role of immunotherapy for chordoma remains under investigation.

The study of the tumor microenvironment, consisting of tumor, immune, vascular, and fibroblast cells (7), has garnered increased recent attention due to advances in immunotherapeutic options. An understanding of the host immune antitumor response can be important in prognostication, tumor phenotyping, and optimization of targeted treatment (8). However, the characterization of chordoma’s complex and heterogeneous tumor immune microenvironment continues to be preliminary (7, 9). Of particular interest in the TIME are the myeloid, T cell, and natural killer cell compartments (10–12). Myeloid cells include macrophages, monocytes, neutrophils, and other cell types that can have a broad array of immunomodulatory effects on the TIME (13). Furthermore, myeloid cells can differentiate into myeloid-derived suppressor cells (MDSC) which can have profound immunosuppressive disruption of T cell mediated tumor immunity (13, 14). Amongst T cells, CD4^+^ helper and CD8^+^ cytotoxic T cells enact immune surveillance, recognition, and destruction of tumor cells while regulatory T cells (T_reg_) suppress anti-tumor immune responses (15). In contrast to T cells, natural killer (NK) cells do not depend on antigen presentation for their activity and can work in concerted effort with antibodies and T cells to enact tumor cell destruction (16).

Harnessing a patient’s immune system to maximize therapeutic efficacy and limit treatment toxicity is a promising research direction for many treatment-resistant malignancies. Immune checkpoint blockade is one such method that may prove beneficial for chordoma (8). The programmed cell death receptor/programmed cell death ligand 1 (PD-1/PD-L1) axis is a known pathway exploited by neoplasms to evade immune surveillance (17, 18), inhibition of which has demonstrated clinical efficacy in melanoma and malignancies of the lung (19, 20). In chordoma specifically, tumor infiltrating lymphocyte (TIL) PD-1 expression has been associated with worsened local recurrence free survival (21). This notion substantiates a current phase II clinical trial underway for nivolumab, a monoclonal antibody against PD-1, in combination with relatlimab, another immune checkpoint inhibitor, for the treatment of recurrent, advanced or metastatic chordoma (NCT03623854). Derivation of additional therapeutic targets for the treatment of chordoma must stem from a better understanding of its TIME. Future immunotherapeutic chordoma clinical trial design would also significantly benefit, as prior trials in breast cancer (22) and melanoma (23) have, from precise evaluation of pre- versus post- treatment tumor-host immune interactions.

Multispectral immunofluorescence (MIF) is an advanced technique that involves staining of tissue specimens with panels of multiple simultaneous antibody markers and allows for appraisal of the high-resolution images. Single cell analyses of these images enable cellular characterization by surface phenotype into tumor compartment (i.e. tumor and stroma), precise immune cell and tumor cell quantification, and evaluation of spatial arrangements among these. Herein, we describe the first comprehensive interrogation of the chordoma TIME including the myeloid cell, T cell, and NK cell compartments, using MIF. We hypothesized that myeloid, T cells, and NK cells would be identifiable within the chordoma TIME, and that effector cells may exhibit restricted tumor penetrance while cells with potentially immunosuppressive activity may be seen within closer proximity to chordoma cells.

## Methods

### Patient population

Ten chordoma specimens were obtained through a Johns Hopkins Institutional Review Board approved study (IRB00227737) and each patient provided written informed consent. Ten chordoma specimens were also obtained from the Chordoma Foundation Biobank and studied following National Institutes of Health Institutional Review Board exemption. Additionally, a tumor microarray (TMA) of 37 anonymized chordoma samples was obtained from the NIH Department of Pathology, totaling a sample size of 57 chordomas. Of these, clinical data could be acquired for the 20 patients from the Chordoma Foundation and Johns Hopkins University. A retrospective chart review of chordoma patients at Johns Hopkins University was conducted. Clinical variables collected included patient demographics (age, sex, race, and ethnicity), anatomic site of tumor origin (skull base, spine, sacrum/coccyx), disease stage, tumor resection extent, tumor grade, treatment regimen (chemotherapy and radiotherapy), and last documented clinical status.

### Tissue preparation

Slides were first baked at 60°C for 30 minutes and soaked in Bond Dewax Solution (Leica Biosystems, #AR9222) at 72°C followed by rehydration with 100% ethanol. Leica BOND Rx autostainer (Leica Biosystems Melbourne Pty Ltd, Melbourne, Australia) was used for deparaffinization and staining of all tissue.

### Panel validation

The dilution of each antibody specific to formalin fixed paraffin embedded (FFPE) chordoma tissue type was determined first using monoplex immunohistochemistry (IHC) and then monoplex immunofluorescence (IF). Staining strength and specificity for the marker of interest with IHC, carried out using chromogen 3’-3’ diaminobenzidine tetrahydrochloride hydrate (DAB) detection (BOND Polymer Refine Detection, Leica Biosystems, New Castle Upon Tyne, UK; #DS9800), determined the dilution to be used as a starting point for IF optimization. IHC positive control tissue, either normal tonsil or normal lung tissue (4-5µm sections), was chosen based on previously identified protein of interest expression, as listed in the Human Protein Atlas (proteinatlas.org) (24). Unstained chordoma tissue (4-5µm sections) served as a negative control for all validation steps. Once optimal IF dilutions were established, multiple combinations of antibodies were tested to determine the most appropriate staining order for the final creation of each multispectral immunofluorescence (MIF) panel (**Supplementary Table 1**).

The myeloid cell panel was comprised of antibodies against CD15 (BD Biosciences [HI98], #555400; 1:1500), CD68 (Invitrogen [KP1], #MA5-13324; 1:1000), CD11b (Abcam [EPR1344], #ab133357; 1:5000), CD14 (Abcam [EPR3653], #ab133335; 1:1000), HLA-DR (Abcam [TAL 1B5], #ab20181; 1:1200), and cytokeratin (Santa Cruz [AE1/AE3], #sc-81714; 1:400). Staining was completed in this order. The myeloid panel allowed for the recognition of pan macrophages, monocytes, and polymorphonuclear (PMN) leukocytes. Cells highly positive for CD68 were categorized as pan macrophages. Tumor associated macrophages (TAMs) express a number of macrophage-specific markers, among these CD68, a heavily glycosylated type I transmembrane glycoprotein (25, 26). CD68 was chosen to identify pan macrophages in this study in keeping with prior investigations of TAMs for the purposes of generalizability and because CD68 has been shown as a good prognostic indicator of cancer patient survival (27–30). Monocyte phenotypes were CD11b^+^/CD14^+^/CD15^-^/HLA-DR while PMN phenotypes were CD11b^+^/C14^-^/CD15^+^/HLA-DR^-^. In the literature, monocyte myeloid derived suppressor cells have been defined as CD11b^+^CD14^+^ CD15^−^HLA-DR^−/^lo. Conversely, PMN myeloid derived suppressor cells have been defined as CD11b^+^CD14^−^CD15^+^ (31, 32). These established definitions served as the authors’ rationale for identifying myeloid cell subtypes as described above. However, we acknowledge that in the absence of functional assays one cannot definitely determine whether monocytes and PMNs meeting the phenotypic criteria direct a suppressive response.

The T cell panel consisted of antibodies against CD4 (Abcam [EPR6855], #ab133616; 1:1000), CD8 (Abcam [EPR10640(2)], #ab215041; 1:2000), FOXP3 (Invitrogen [SP97], #MA5-16365; 1:200), PD-1 (Abcam [EPR4877(2)], #ab137132; 1:750), Ki67 (Ventana [30-9], #790-4286; 1:1), and cytokeratin (Santa Cruz [AE1/AE3], #sc-81714; 1:400). Staining was completed in this order. This T cell panel revealed the presence of T-regulatory cells, CD4^+^ T-helper cells, and CD8^+^ cytotoxic T cells. T-regulatory cells were simultaneously positive for CD4 and FOXP3 markers, while non-T-regulatory cells were FOXP3 negative. CD4^+^ T-helper cell phenotypes were CD4^+^ and CD8^-^/FOXP3^-^, while CD8^+^ cytotoxic T cell phenotypes were CD8^+^ and CD4^-^/FOXP3^-^. Positive Ki67 staining denoted proliferating cells. PD-1 stain allowed for identification of PD-1^+^ T cells.

The NK cell panel was comprised of antibodies against CD3 (Abcam [SP7], #ab16669; 1:300), CD56 (NCAM1) (Sigma Aldrich [MRQ-42], #156R-94; 1:250), CD16 (Abcam [SP175], #ab183354; 1:150), Granzyme B (Abcam [EPR8260], #ab134933; 1:200), and cytokeratin (Santa Cruz [AE1/AE3], #sc-81714; 1:400). Staining was completed in this order. CD16^+^/CD56^+^/CD3^-^ and a decreased size threshold relative to tumor cells was the phenotype used to identify NK cells. Granzyme B positivity denoted activated NK cells.

Similar to the validation stages, positive and negative controls included normal human tonsil or lung tissue and unstained sections lacking primary antibody, respectively. All slides were counterstained with 4′,6-diamidino-2-phenylindole (DAPI) to identify cell nuclei. Cytokeratin distinguished chordoma tumor cells in all panels.

### Staining and scanning

Standardized staining protocols (Perkin Elmer platform), using the Leica Bond Rx automated system described above, were completed with 4µm FFPE sections of chordoma tissue. Heat induced epitope retrieval (HIER) was executed for all antibodies except CD15 by heating slides to 95°C and treating with paired BOND Epitope Retrieval (ER) solutions, either citrate-based ER1 (Leica Biosystems, #AR9961) or EDTA-based ER2 (Leica Biosystems, #AR9640). Primary antibody was applied for 60 minutes, followed by secondary HRP-conjugated antibody for 30 minutes, and finalized by fluorescent signal amplification for every protein target. OPAL (Akoya Biosciences) multiplex kit consisting of OPAL-480, 520, 570, 620, 690, 780 conjugates allowed for the evaluation of up to six colors simultaneously. With the exception of Opal 780 made at 1:50 dilution, all opals were made at a 1:150 dilution. Opal 780 was combined with TSA-DIG at a 1:100 dilution. After staining, slides were cover slipped with the Leica CV5030 automated glass cover slipper (Leica Biosystems, Nussloch, Germany Ltd) and scanned at 40x magnification with suitable exposure times using PerkinElmer Vectra Polaris for the creation of high-resolution digital images. One slide stained with the myeloid panel was excluded due to technical issues with slide scanning and three tumors stained with the NK panel on the tumor microarray slide were excluded due to overexposure with scanning parameters best suited to the greatest number of tumors.

### Image analysis

Images were analyzed using the HALO^®^ (Indica Labs, Albuquerque, NM, USA) platform v3.3, which allows for diverse cell type quantification, identification of marker of interest co-localization, and cellular spatial relation analysis. First, slides were annotated into tumor core and stroma using the HALO^®^ random forest classifier based on cytokeratin staining. Histologic areas of bone, bone marrow, and blood vessels, identified using hematoxylin and eosin (H&E) stains, along with auto fluorescent tissue subsections, were manually excluded for analysis. Cell size, nuclear to cytoplasmic ratio, nuclear segmentation, and individual biomarker fluorescence intensity thresholds were set to denote cells of each phenotype of interest. These were calibrated for each independent specimen to account for variability in staining uptake. Quantification analyses were run using the HALO^®^ Highplex FL analysis algorithm v4.1.3. Nearest neighbor analyses, to determine the average distance and number of unique neighbors between two cell populations, and proximity analyses, for calculation of the number of cells within a 20µm or 100µm distance of a given cell of interest, were conducted. These were used to investigate distances of helper T cells, cytotoxic T cells, T-regulatory cells, pan macrophages, monocytes, and PMNs to chordoma cells. Spatial relationships between T-regulatory cells and cytotoxic T cells were also evaluated. HALO^®^ density heat maps of all immune cell compartments were created to visually compare immune infiltrate patterns.

### Statistical analysis

Immune cell densities and spatial relationships were represented with box and whisker plots and histograms, respectively. Wilcoxon signed rank tests were used to detect statistical differences between dependent groups of tumors versus stroma immune cell density. A Wilcoxon signed rank test was also used to evaluate the difference between T-regulatory cell distances to cytotoxic T cells by proliferation status. Two-way ANOVA analysis was used to compare immune cell densities among groups by anatomic site of origin and disease stage. A *p* value significance threshold of <0.05 was employed in all cases. All statistics were conducted and graphs prepared using GraphPad Prism version 9.2.

## Results

**Figure 1:**
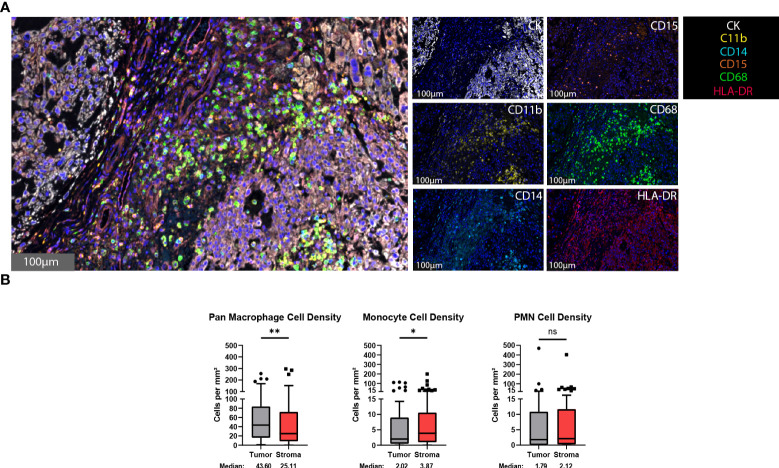
Myeloid cells are found within the chordoma tumor immune microenvironment. **(A)** Representative photomicrographs from one chordoma of merged and single-color immunofluorescence images assessing the presence of myeloid cells with a validated panel of six biomarkers, CD11b (yellow, Opal 570), HLA-DR (red, Opal 690), CD14 (turquoise, Opal 480), CD15 (orange, Opal 620), CD68 (green, Opal 520), and CK (white, Opal 780). Multispectral immunofluorescence images are counterstained with DAPI. High expression of CD68 identified pan macrophages. Co-localization of CD11b and CD14 without CD15 and HLA-DR identified monocytes. Co-localization of CD11b and CD15 without CD14 and HLA-DR identified PMNs. Expression of CK identified chordoma tumor cells. **(B)** Quantification of pan macrophages, monocytes, and PMNs per mm^2^ in chordoma tumor and stroma (n = 56 individual tumors). Wilcoxon signed rank tests with p value threshold of <0.05 were used to compare tumor and stroma myeloid cell infiltrate. *p ≤ 0.05, **p ≤ 0.01, ns, non significant. CK, cytokeratin; DAPI, 4′,6-diamidino-2- phenylindole; PMN, polymorphonuclear cells.

The presence of myeloid cells, particularly a myeloid-derived suppressor cell subset, within the tumor microenvironment has been associated with immunosuppression and a decreased clinical immunotherapy response (32–34). To identify myeloid cells within the chordoma tumor microenvironment, chordoma tissue sections were stained using an automated multispectral immunofluorescence system with antibodies against C11b, HLA-DR, CD14, CD15, CD68, and cytokeratin (CK) (**Figure 1A**) followed by digital scanning, image annotation, and cellular analysis (**Supplementary Figure 1**). Significant tumor parenchymal myeloid cell infiltration was noted. The highest expressing CD68 cells were classified as pan macrophages, and cytokeratin positive cells as chordoma tumor cells. Myeloid cell quantification analysis revealed that macrophages infiltrated the tumor parenchyma at a significantly higher density than stroma (median 43.60 cells per mm^2^ in tumor, median 25.11 cells per mm^2^ in stroma, *p* = 0.006) and made up the majority of myeloid cells found within the chordoma tumor microenvironment (**Figure 1B**). Co-localization of CD11b and CD14 in the absence of CD15 and with low HLA-DR identified monocytes. There was a slightly greater monocyte cell density identified in the stroma as compared to tumor (median 2.02 cells per mm^2^ in tumor, median 3.87 cells per mm^2^ in stroma, *p* = 0.036) (**Figure 1B**). Co-localization of CD11b and CD15 in the absence of CD14 and with low HLA-DR identified PMN-like cells. PMN-like cell density was comparable between tumor and stroma (median 1.79 cells per mm^2^ in tumor, median 2.12 cells per mm^2^ in stroma, *p* = 0.378) (**Figure 1B**). Myeloid cell density differences between tumor and stroma are graphed using mean ± standard error of the mean (SEM) in **Supplementary Figure 2**.

**Figure 2:**
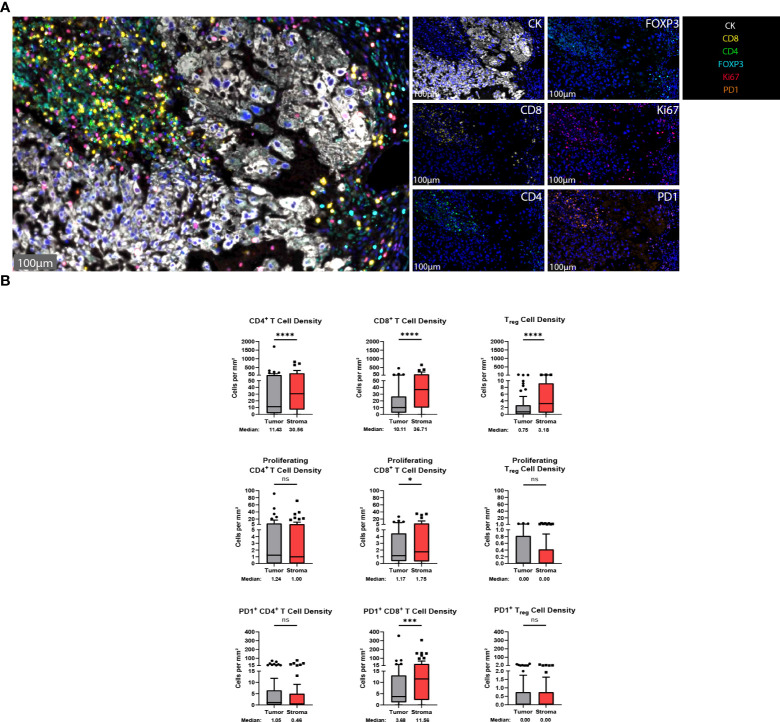
T cells are found in abundance within the chordoma tumor immune microenvironment, most often within the stroma. **(A)** Representative photomicrographs from one chordoma of merged and single-color immunofluorescence images assessing the presence of T cells with a validated panel of six biomarkers, CD8 (yellow, Opal 570), CD4 (green, Opal 520), FOXP3 (turquoise, Opal 480), Ki67 (red, Opal 690), PD1 (orange, Opal 620), and CK (white, Opal 780). Multispectral immunofluorescence images are counterstained with DAPI. Expression of CD4 without CD8 and FOXP3 identified CD4^+^ T helper cells, while expression of CD8 without CD4 and FOXP3 identified CD8^+^ cytotoxic effector T cells. Co-localization of CD4 and FOXP3 without CD8 identified T_regs_. T cells with positive Ki67 nuclear expression were proliferating, and PD1 positivity denoted PD1^+^ T cells. Expression of CK identified chordoma tumor cells. **(B)** Quantification of CD4^+^ T cell, CD8^+^ T cell, and T_reg_ cell density (per mm^2)^, as well as proliferating and PD-1^+^ subcategories of these are compared between chordoma tumor and stroma (n = 57 individual tumors). Wilcoxon signed rank tests with p value threshold of <0.05 were used to compare tumor and stroma T cell infiltrate. *p ≤ 0.05, ***p ≤ 0.001, ****p ≤ 0.0001, ns, non significant. CK, cytokeratin; DAPI, 4′,6-diamidino-2- phenylindole; PD1, programmed cell death protein 1.

T cells are vital players to the antitumor response and represent some of the most important targets for immunotherapeutics to date (10). To identify T cells within the chordoma tumor immune microenvironment, tissue sections were stained with a panel of antibodies against CD4, CD8, FOXP3, Ki67, PD-1, and CK (**Figure 2A**). Staining with the validated T cell panel showed grossly appreciable T cell tumor parenchymal exclusion with restriction of most T cells to stromal tissue (**Figure 2A**). FOXP3 negative CD4^+^ cells were identified as helper T cells, co-localization of CD4 and FOXP3 in the absence of CD8 identified T_regs_, and CD8^+^ cells were identified as T cells with potential cytotoxic activity against chordoma cells. T cell quantification analysis objectively revealed significantly more T cells per mm^2^ in stroma as compared to tumor for all CD4^+^ T cells (median 11.43 and 30.56 cells per mm^2^ in tumor and stroma, respectively, *p* < 0.0001), CD8^+^ T cells (median 10.11 and 36.71 cells per mm^2^ in tumor and stroma, respectively, *p* < 0.0001), and T_regs_ (median 0.75 and 3.18 cells per mm^2^ in tumor and stroma, respectively, *p* value < 0.0001) (**Figure 2B**). Proliferating CD8^+^ T cells (median 1.17 and 1.75 cells per mm^2^ in tumor and stroma, respectively, *p* = 0.011) and PD1^+^ CD8^+^ T cells (median 3.68 and 11.56 cells per mm^2^ in tumor and stroma, respectively, *p* = 0.0001) were also significantly more abundant in chordoma stroma. Although proliferating CD4^+^ T cells (median 1.24 and 1.00 cells per mm^2^ in tumor and stroma, respectively, *p* = 0.888) and PD1^+^ CD4^+^ T cells (median 1.05 and 0.46 cells per mm^2^ in tumor and stroma, respectively, *p* = 0.180) were slightly more abundant in the tumor parenchyma, these small differences failed to reach statistical significance. Only a minor subset of samples contained proliferating T_regs_ (median 0.00 cells per mm^2^ in tumor and stroma) and PD1^+^ T_regs_ (median 0.00 cells per mm^2^ in tumor and stroma) (**Figure 2B**). Similarly, Ki67^+^, or proliferating CD4^+^ and CD8^+^ T cells, made up small proportions, less than 20%, of these cell types. Of note, the PD1^+^ CD8^+^ T cell subcategory represented over one third of cytotoxic T cells. T cell density differences between tumor and stroma are graphed using mean ± standard error of the mean (SEM) in **Supplementary Figure 3**.

**Figure 3:**
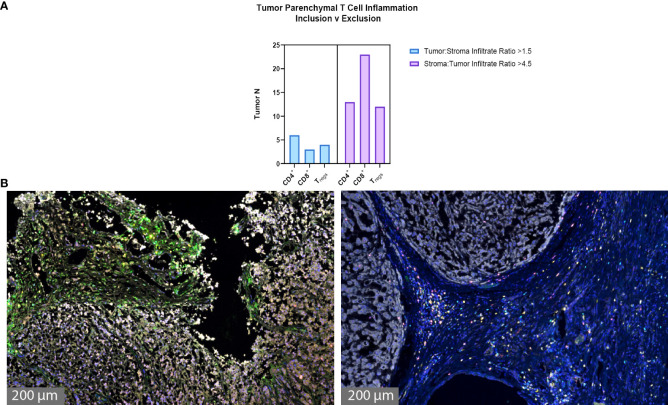
Chordoma tumor parenchymal T cell inclusion versus exclusion. T cell compartment inflammation in chordoma more commonly takes on a tumor parenchymal exclusion, rather than inclusion, pattern (n = 57 individual tumors). **(A)** A tumor:stroma T cell infiltrate ratio >1.5 defined substantial tumor parenchymal inclusion. A stroma:tumor T cell infiltrate ratio >4.5 defined substantial tumor parenchymal exclusion. **(B)** Representative chordoma samples with heavy tumoral infiltrate (left) versus heavy stromal infiltrate (right).

The degree of tumor parenchymal T cell inclusion versus exclusion was assessed (**Figures 3A, B**). A ratio of >1.5 tumor to stroma T cell infiltrate defined substantial T cell inclusion whereas a ratio of >4.5 stroma to tumor T cell infiltrate defined substantial T cell exclusion. **Figure 3A** shows high parenchymal CD4^+^ T cell inclusion in six samples, high CD8^+^ T cell inclusion in three samples, and high T_reg_ inclusion in four samples. However, notable tumor parenchymal exclusion was seen at higher rates, with 13 samples meeting criteria for high CD4^+^ T cell exclusion, 23 samples for high CD8^+^ T cell exclusion, and 12 samples for high T_reg_ exclusion. Thus, chordoma T cell compartment inflammation more commonly takes on a tumor parenchymal exclusion pattern, most markedly among cytotoxic T cells.

**Figure 4:**
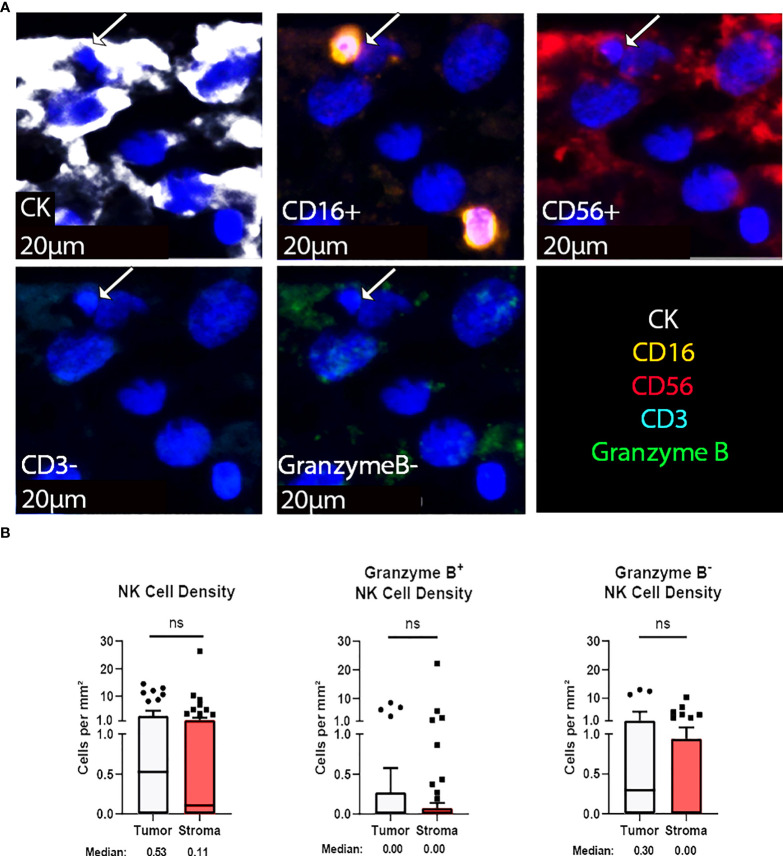
NK cells are sparsely found within the chordoma tumor immune microenvironment. **(A)** Representative photomicrographs from one chordoma of merged and single-color immunofluorescence images assessing the presence of NK cells with a validated panel of five biomarkers, CD16 (orange, Opal 620), CD56 (red, Opal 690), CD3 (turquoise, Opal 480), Granzyme B (green, Opal 520), and CK (white, Opal 780). Multispectral immunofluorescence images are counterstained with DAPI. Co-localization of CD16 and CD56 without CD3 and a decrease size threshold compared to tumor cells identified NK cells. Expression of Granzyme B identified Granzyme B^+^ or Granzyme B^-^ NK cells. Expression of CK identified chordoma tumor cells. **(B)** Quantification of NK cells overall, Granzyme B^+^, and Granzyme B^-^ NK cells per mm^2^ in chordoma tumor and stroma (n = 54 individual tumors). Wilcoxon signed rank tests with p value threshold of <0.05 were used to compare tumor and stroma NK cell infiltrate. ns, non significant. NK cell, natural killer cell; CK, cytokeratin; DAPI, 4′,6-diamidino-2- phenylindole.

Natural killer (NK) cells are viable therapeutic targets known to kill adjacent cells independently of antigen presentation and have been shown by our group to effectively act against chordoma tumor cells in an inducible fashion (11, 16). CD56, also known as neural cell adhesion molecule, is a common marker for NK cells. However, we found that a significant proportion of chordoma cells express CD56 as previously reported (35). We therefore defined NK cells by a decreased size threshold as compared to larger chordoma cells along with CD16, CD56 co-localization in the absence of CD3. Granzyme B positivity revealed active NK cells. NK cells were sparsely observed in chordoma tissue (**Figure 4A**). Quantification uncovered slightly greater, albeit non-significant, tumor NK cell infiltration (median 0.52 cells per mm^2^ in tumor, median 0.11 cells per mm^2^ in stroma, *p* = 0.085) with similar patterns detected by Granzyme B^-^ subcategorization (median 0.30 cells per mm^2^ in tumor, median 0.00 cells per mm^2^, *p* = 0.056) (**Figure 4B**). Few NK cells expressed Granzyme B overall (median 0.00 cells per mm^2^ in tumor and stroma), although a subset of five tumors were identified that expressed >2 Granzyme B^+^ NK cells per mm^2^ (**Figure 4B**).

**Figure 5:**
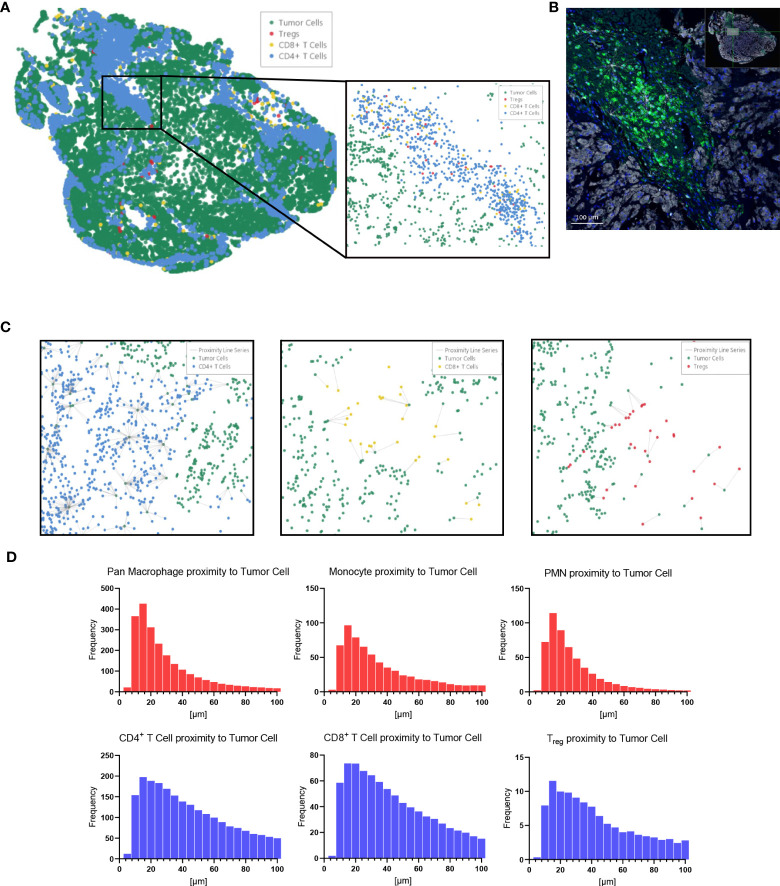
HALO^®^ spatial proximity analyses of immune cells to chordoma cells demonstrate that T cells are nearer than myeloid cells to tumor cells. **(A)** Sacral chordoma specimen with three categories of T cells, CD4^+^ T cells, CD8^+^ T cells, and T_regs_ shown in blue, yellow, and red respectively. Tumor cells are shown in green. A segment of heavy T cell infiltrate is magnified. **(B)** Real time proximity analysis between CD4^+^ T cells (green, Opal 520) and chordoma cells (white, Opal 780) with bars measuring distance between these superimposed on the image in white. **(C)** Proximity line series between T cells and chordoma tumor cells. **(D)** Proximity analysis histograms of myeloid cells (top row, red) and T cells (bottom row, blue) to chordoma tumor cells within a 100µm radius by progressive segments of 20µm bands (n = 56 individual tumors).

**Figure 6:**
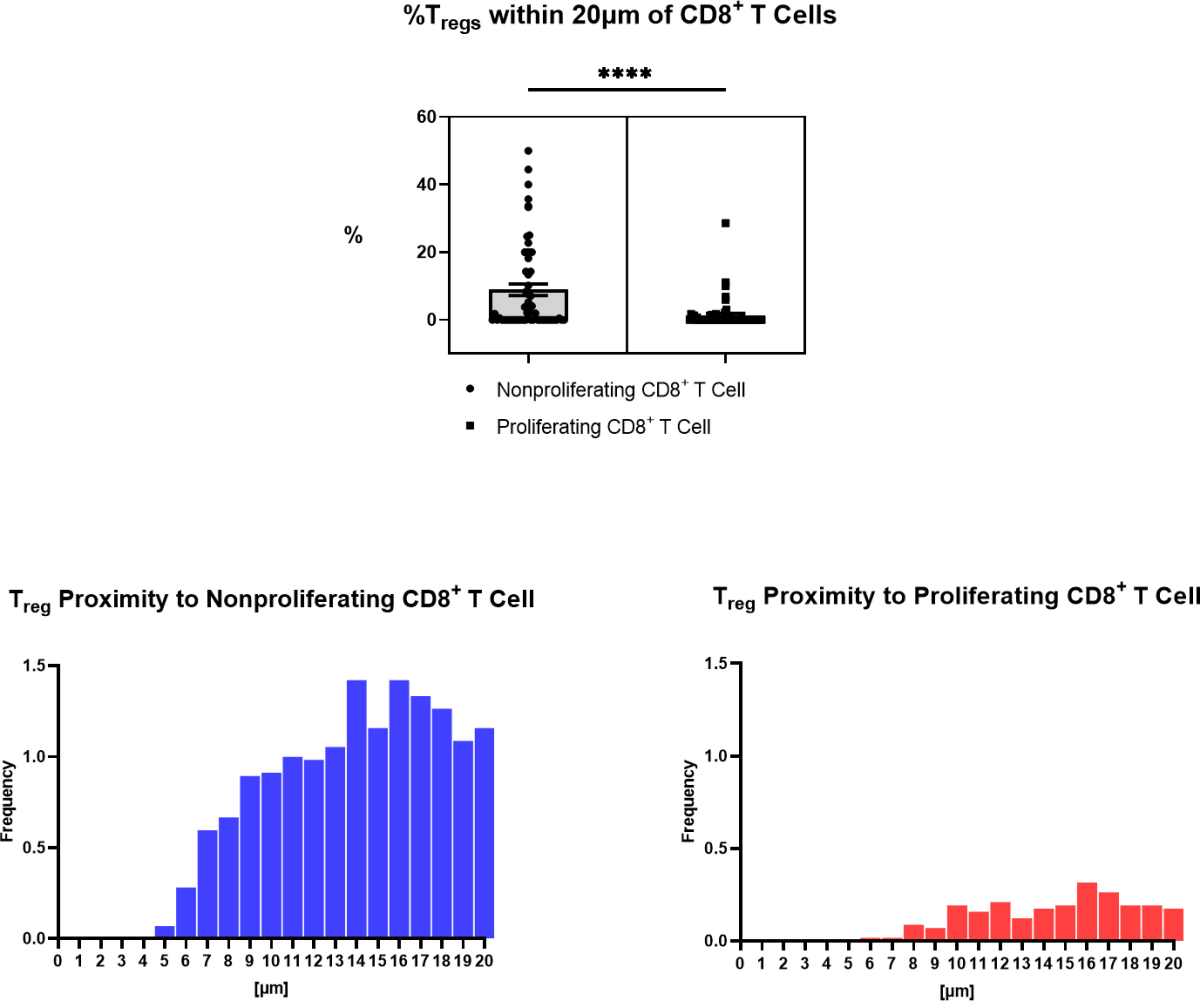
Spatial characterization of distance between Tregs to proliferating and nonproliferating CD8+ T cells in chordoma tumor slides (n = 57). Percentage of Tregs within 20µm of nonproliferating CD8+ T cells (mean 8.90 ± SEM 1.74) is significantly greater than that within 20µm of proliferating CD8+ T cells (mean 1.34 ± SEM 0.57) (p < 0.0001), shown above. A Wilcoxon signed rank test with a p value threshold of <0.05 was used. ****p ≤ 0.0001. Proximity analysis histograms of Tregs within a 20µm radius of nonproliferating CD8+ T cells (left, blue) and proliferating CD8+ T cells (right, red) by progressive segments of 1µm bands, shown below.

Immune cell spatial analysis using the HALO^®^ (Indica Labs) v3.3 platform allowed for a greater understanding of spatial relationships between immune cells and tumor cells within the chordoma tumor immune microenvironment (**Figures 5A–C**). The majority of myeloid cells and T cells were found within 30µm and 50µm of chordoma cells, respectively (**Figure 5D**). Myeloid cell mean distance to chordoma cells was comparable for all three cell types, pan macrophage: 36.55µm ± 25.70µm, PMN-like: 43.59µm ± 37.15 µm, monocyte: 50.67µm ± 45.54µm. Mean distance to chordoma cells was likewise similar among all T cells, CD4^+^ T cells: 97.40µm ± 185.07µm, CD8^+^ T cells: 92.52µm ± 167.04µm, and T_regs_: 114.66µm ± 165.67µm. The distance between T cells and tumor cells was consistently greater than the distance between myeloid cells and tumor cells. Similarly, the mean percentage of myeloid cells identified within 100µm of chordoma cells (PMN: 90.68% ± 19.79%, monocyte: 86.66% ± 23.44%, pan macrophage: 93.34% ± 11.74%) was greater than the mean percentage of T cells identified within 100µm of chordoma cells (CD4^+^ T cells: 81.40% ± 26.22%, CD8^+^ T cells: 80.49% ± 26.65%, T_regs_: 62.76% ± 33.15%). The T_reg_ phenotype is known to abrogate the CD8^+^ T cell cytotoxic response and inhibit effector T cell expansion (36, 37). Thus, it is important to characterize the spatial relationship that exists between T_regs_ and effector cytotoxic T cells within the chordoma tumor immune microenvironment. The mean percentage of T_regs_ residing within 20µm of nonproliferating CD8^+^ T cells (8.90 ± 13.05) was significantly greater (*p* < 0.0001) than that residing within 20µm of proliferating CD8^+^ T cells (1.34 ± 4.28) (p < 0.001) (**Figure 6**). These data indicate that T_regs_, consistent with known immunosuppressive capacity in other solid tumor types, may be abrogating the proliferative capacity of cytotoxic T cells. Density heat maps of a representative chordoma specimen illustrated similar immune cell infiltration patterns among myeloid cells, T cells, and NK cells (**Supplementary Figure 4**).

**Figure 7:**
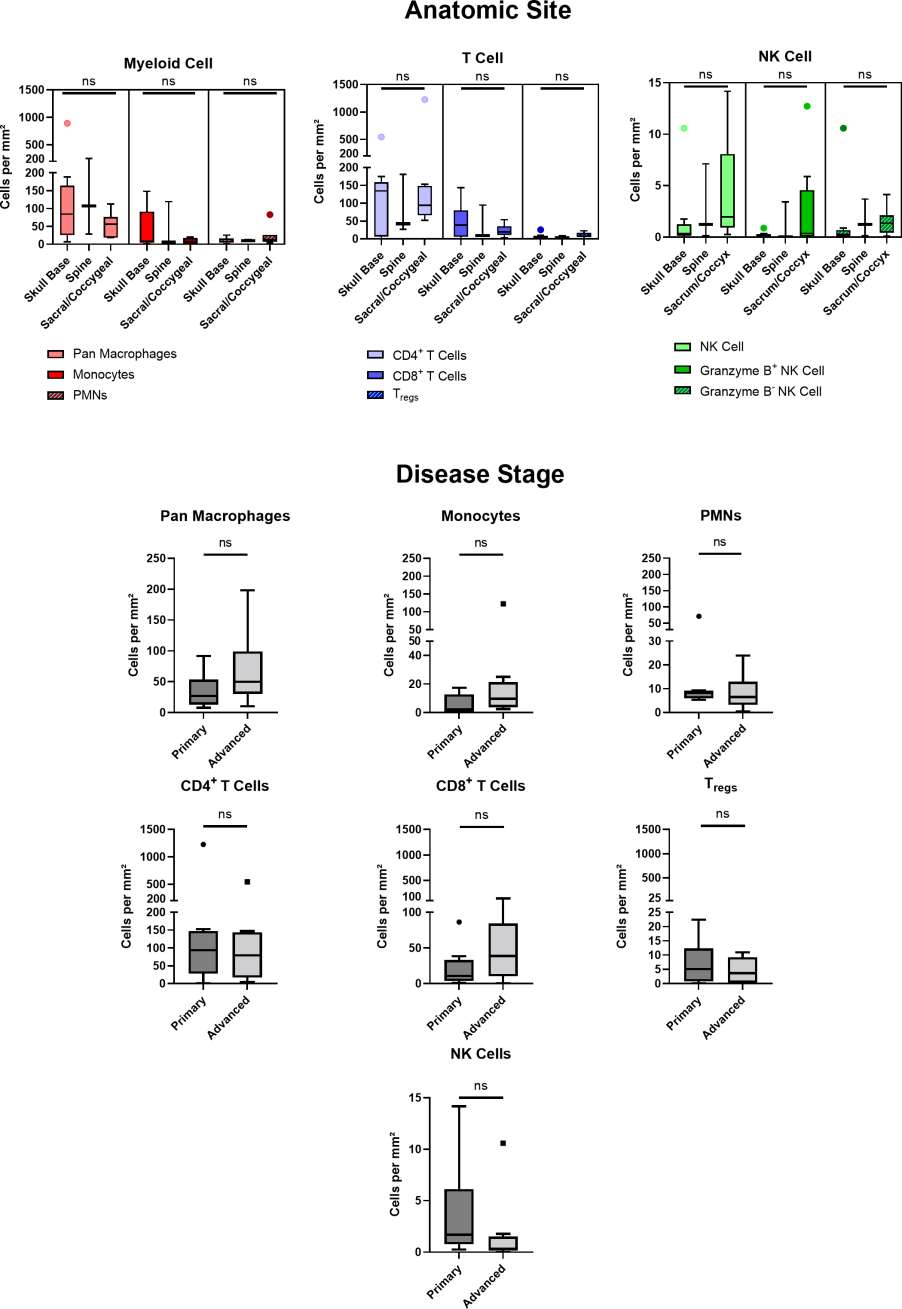
Chordoma myeloid, T cell, and NK cell density does not significantly differ by anatomic site. Immune infiltrate differences among chordomas (n = 20) originating from skull base (n = 9), spine (n = 3), or sacrum/coccyx (n = 8) were assessed with two-way ANOVA analysis using a p value threshold of <0.05. Chordoma myeloid, T cell, and NK cell density does not significantly differ by disease stage. Immune infiltrate differences between chordoma patients (n = 20) with primary and advanced disease were assessed with Mann Whitney tests using a p value threshold of <0.05. Advanced disease was defined as including cases of locally progressive and locally advanced, destructive disease. ns, non significant. NK cell, natural killer cell.

Of 20 patients with available clinical data, 40% were of female sex, 94% of Caucasian race, and 15% of Hispanic ethnicity. The mean patient age was 49 ± 16 years. Patient clinical data is delineated in **Table 1**. Interestingly, myeloid cell, T cell, and NK cell density did not significantly differ among tumors originating from skull base, spine, or sacral anatomic subsites (**Figure 7**). No significant differences were appreciated in the quantity or character of chordoma immune infiltrate of patients with primary or locally advanced disease (**Figure 7**). No significant differences in immune cell infiltrate were appreciated in tumors arising from patients ≤ 35 years old compared to those of patients >35 years old (**Supplementary Figure 5**).

**Table 1:** Patient Clinical Data.

	N (%)
Anatomic Site of Origin	
Skull Base	9 (45%)
Spine	3 (15%)
Sacral/Coccygeal	8 (40%)
Disease Stage	
Primary	9 (45%)
Advanced	9 (45%)
Recurrent	2 (10%)
Extent of Resection	
Gross Total/Near Gross Total	13 (65%)
Partial	1 (5%)
Unavailable	6 (30%)
Therapeutic Regimen	
Pre- or postoperative radiotherapy	11 (55%)
Chemotherapy	0 (0%)

Clinical data of patients diagnosed with chordoma included in the study (n = 20). Data is shown as n(%).

## Discussion

Chordoma is a rare tumor of the skull base and axial skeleton with a well-established radiotherapy resistance and a strong propensity for recurrence for which immunotherapeutic strategies could play an important role. To adeptly set the stage for future immunotherapy investigations and clinical trial design, we appraised the myeloid, T cell, and NK cell compartments of the chordoma TIME using multispectral immunofluorescence. Our data demonstrated a strong myeloid tumor parenchymal infiltrate, a predominant T cell parenchymal exclusion profile, a spatial analysis suggestive of T_reg_ immunosuppression, and the presence of a small number of non-activated NK cells. Similar to extensively validated data in other solid tumor types, these results suggest that therapeutic strategies aimed at abrogating myeloid cell or T_reg_ tumor trafficking or immunosuppression in addition to direct activation of cytotoxic T cells may be warranted.

In this study, CD68^+^ pan macrophages more significantly infiltrated chordoma tumor parenchyma than stroma, unlike trends appreciated in the T cell compartment, and all myeloid cell types identified were within notably closer proximity to chordoma tumor cells than T cells. Potent macrophage tumor penetrance and myeloid cell proximity to target cells points to a potential immunosuppressive myeloid cell role within the chordoma TIME. Myeloid-derived suppressor cells (MDSCs) are known to foster neoplasm escape of immune-mediated killing through inhibition of T cell and NK cell function (38). Monocytic-MDSCs have been defined as HLA-DR ^low/-^/CD14^+^/CD15^-^/CD11b^+^ and have been associated with poor survival in non-small-cell lung carcinoma (NSCLC) (32). PMN-MDSC have been defined as HLA-DR ^low/-^/CD14^-^/CD15^+^/CD11b^+^ (32). We employed these corresponding phenotypes to identify monocytes and PMN-like cells. Use of archived tissue for this analysis precluded the ability to functionally determine the suppressive capacity of these cells, but these phenotypes have been definitively linked to immunosuppressive capacity in other tumor solid types. In head and neck squamous cell carcinoma, the colony stimulating factor 2 (CSF-2) gene encoding granulocyte-macrophage colony-stimulating factor (GM-CSF) expression, linked to MDSC, was strongly associated with poor overall survival in anti-PD-1 treated recurrent and metastatic patients, and CD34, an MDSC marker, was increased in tumors without derived therapeutic benefit (34). Another study by Karpathiou et al., demonstrated that high levels of infiltrating tumor-associated-macrophages (TAMs) were linked to poor induction chemotherapy response, corroborated by Sugimura et al. in the study of esophageal cancer (39, 40). High TAM infiltrates have been correlated with worsened prognosis in a variety of cancers, including breast, urogenital, gastric, ovarian, thyroid, and prostate neoplasms (41). Sun et al. demonstrated in oral and lung squamous cell carcinoma that small molecule inhibition of C-X-C Motif Chemokine Receptor 2 (CXCR2), important to neutrophil chemotaxis, with SX-682 hindered tumor growth in the setting of PD-axis immune checkpoint inhibition and adoptive T cell transfer therapy (33). This substantiates the strategy of abrogation of myeloid cell trafficking into tumors to enhance T cell effector efficacy and T cell-based immunotherapy. Although further study of the chemokine profiles produced by chordoma cells that result in myeloid infiltration are needed, such strategies may be applied to chordoma in combination with T cell based immunotherapeutic approaches (13, 38).

In this study, we found that T cells were predominantly localized in the stroma and largely excluded from the tumor parenchyma (**Figures 2** **,** **3**). As previous studies of the head and neck squamous cell carcinoma TIME have reported that high lymphocytic tumor infiltration carries favorable prognostic significance and is associated with positive predictive response to induction chemotherapy (39, 42), strategies to increase tumor parenchymal infiltration may be of benefit for chordoma. Additionally, T_reg_ cells were found in closer proximity to non-proliferating CD8^+^ T cells, suggesting that T_reg_ cells may be playing a significant immunosuppressive role in chordoma (**Figure 6**). Thus, targeting T_reg_ cells may also aid in improving T cell activation and proliferation. Lastly, few NK cells were found in chordoma tissue and furthermore very few were in an activated state (**Figure 4**). This suggests that strategies to increase NK cell proliferation and activation such as with interleukin-15 superagonism, or adoptive NK cell therapies may be beneficial for chordoma as previously shown in *in vitro* studies (16). Collectively, this study suggests that future immunotherapy combinations for chordoma should be aimed at decreasing myeloid cell trafficking or suppressive function while enhancing cytotoxic T cell and NK cell killing. Importantly, this study also found a similar immune profile amongst chordoma from each anatomic subsite. A lack of distinct chordoma TIME composition amongst tumors of differing sub-sites and disease stage (**Figure 7**) suggests that chordoma may reasonably be treated with similar approaches and uniformly investigated in clinical trials independent of anatomic site of origin or diagnostic stage.

To our knowledge, this is the first application of multispectral immunofluorescence to skull base chordomas although spinal chordomas (43–45) and a single case of a sacral chordoma (46) have been analyzed with such techniques. Notable strengths of our study include the number of tumors studied and the capacity to study spatial immune cell relationships *in situ* in human tissue samples, obtaining nuance that *in vitro* studies cannot afford, and offering information previously undescribed in the literature. An ability to uniformly stain tumors with antibody panels with an automated staining system (PerkinElmer) controls for variation that accompanies staining by hand. Similarly, uniform analysis methods applied *via* HALO allow for high throughput, precise, and comparable results amongst assorted chordoma samples. Although chordomas are known to be heterogeneous with varying levels of expression of the markers we examined (7), a large sample size aided in achieving a more comprehensive idea of the chordoma TIME than what has previously been described, with the inclusion of myeloid and NK cell compartments.

This study is limited by its retrospective nature and, thus, by restricted access to patient data, particularly treatment regimen and outcome data, for chordoma tissue sections in the tumor microarray. Additionally, recurrent tumors comprised a minor proportion of the cohort available for clinical comparisons (10%, n=2). Due to low statistical power, the authors were unable to include recurrence as an independent category for TIME analysis. Similarly, no metastatic cases were available for inclusion. The study of recurrent and metastatic tumors remain of interest as these have previously been associated with inferior overall survival (47). Therefore, the field would benefit from prospective studies in which differences due to recurrent or metastatic tumor status and the role of radiotherapy on the chordoma TIME and could be further investigated. A limitation of the analysis platform used is an inability to merge or superimpose images of distinct panels (i.e. myeloid cell panel, T cell panel, and NK cell panel) for a more extensive immune cell-to- immune cell spatial relationship analysis. Currently, each panel is restricted to a maximum of six antibodies due to the upper limit of six fluorescent channels of the platform, and thus, myeloid, T cell, and NK cell markers could not be combined within a single panel. These limitations could be overcome with emerging high-plex technologies such as spatial transcriptomics or proteomics. There are also limitations attributable to analysis of a tumor microarray with this method, including the ability to interrogate only a small portion of each individual tumor and an inability to set customized scanning exposure times for every tumor on the slide in efforts to account for varying levels of staining uptake amongst heterogeneous chordoma specimens.

## Conclusion

This study characterizes the human chordoma TIME myeloid cell, T cell, and NK cell composition, making comparisons between tumoral and stromal infiltration and determining spatial relationships between immune cells and tumor cells. We conclude that the presence of myeloid cells, cytotoxic T cells, T_regs_, and NK cells suggest chordoma’s susceptibility to immunotherapeutic approaches. Given findings of increased proximity of myeloid cells to tumor cells in the setting of decreased tumor penetrance by effector T cells, and co-localization of T_regs_ and cytotoxic T cells in the stroma, treatment strategies aimed at restricting the trafficking and function of potentially immunosuppressive myeloid cells and T_regs_ may allow for greater T cell killing of target chordoma cells. The establishment of an optimized chordoma immunofluorescence protocol also affords scientists a powerful tool with which to compare TIME alterations in future immunotherapeutic studies with application to clinical trial design.

## Data availability statement

The raw data supporting the conclusions of this article will be made available by the authors, without undue reservation.

## Ethics statement

Ten chordoma specimens were obtained through a Johns Hopkins Institutional Review Board approved study (IRB00227737) and each patient provided written informed consent. Ten chordoma specimens were also obtained from the Chordoma Foundation Biobank and studied following National Institutes of Health Institutional Review Board exemption. Additionally, a tumor microarray (TMA) of 37 anonymized chordoma samples was obtained from the NIH Department of Pathology, totaling a sample size of 57 chordomas.

## Author contributions

Conceptualization: DL, JG, GG, CA, JH, and NL. Methodology: DL, YR, JK, WL, JG, GG, CA, JH, and NL. Investigation: DL, YR, JK, WL, JG, MM, GG, CA, JH, and NL. Funding acquisition: JH and NL. Writing – original draft: DL, JH, and NL. Writing – review and editing: DL, YR, JK, WL, JG, MM, GG, CA, JH, and NL. All authors contributed to the article and approved the submitted version.

## Funding

This research was supported (in part) by the Intramural Research Program of the NIH, NIDCD. This work was funded by a Translational Immunotherapy Award from the Chordoma Foundation to N. London. This research was made possible through the NIH Medical Research Scholars Program, a public-private partnership supported jointly by the NIH and contributions to the NIH from the Doris Duke Charitable Foundation (DDCF Grant #2014194), the American Association for Dental Research, the Colgate-Palmolive Company, Genentech, Elsevier, and other private donors.

## Acknowledgments

We would like to thank Dr. Wade Chien and Dr. Michael Hoa for critical review of this manuscript.

## Conflict of interest

NL receives research funding from Merck Sharp & Dohme, LLC regarding HPV related sinonasal carcinomas, holds stock in Navigen Pharmaceuticals and was a consultant for Cooltech Inc., none of which are relevant to the present manuscript.

The remaining authors declare that the research was conducted in the absence of any commercial or financial relationships that could be construed as a potential conflict of interest.

## Publisher’s note

All claims expressed in this article are solely those of the authors and do not necessarily represent those of their affiliated organizations, or those of the publisher, the editors and the reviewers. Any product that may be evaluated in this article, or claim that may be made by its manufacturer, is not guaranteed or endorsed by the publisher.

## Supplementary material

Supplementary Figure 1 Chordoma tissue samples (n=57) were stained using the Leica BOND Rx automated system (PerkinElmer protocol), scanned with VectraPolaris, and analyzed using the HALO^®^ (Indica Labs) v3.3 platform (first panel). For analysis, slides were digitally annotated based on cytokeratin stain (white, Opal 780), which distinguished CK^+^ chordoma cells from surrounding CK^-^ stroma (third panel). Immune cell quantification, spatial analysis, and clinical comparisons were completed using the HALO^®^ platform and GraphPad Prism version 9.2 (fourth panel).

Supplementary Figure 2 Myeloid cells are found within the chordoma tumor immune microenvironment. Quantification of pan macrophages, monocytes, and PMNs per mm2 in chordoma tumor and stroma (n= 56 individual tumors) presented in formatted here as mean ± standard error of the mean. Wilcoxon signed rank tests with p value threshold of <0.05 were used to compare tumor and stroma myeloid cell infiltrate. *p ≤ 0.05, **p ≤ 0.01,ns, non significant. CK, cytokeratin; DAPI, 4′,6-diamidino-2- phenylindole; PMN, polymorphonuclear cells.

Supplementary Figure 3 T cells are found in abundance within the chordoma tumor immune microenvironment, most often within the stroma. Quantification of CD4^+^ T cell, CD8^+^ T cell, and T_reg_ cell density (per mm^2)^, as well as proliferating and PD-1^+^ subcategories of these are compared between chordoma tumor and stroma (n= 57 individual tumors) presented in formatted here as mean ± standard error of the mean. Wilcoxon signed rank tests with p value threshold of <0.05 were used to compare tumor and stroma T cell infiltrate. **p ≤* 0.05, ****p ≤* 0.001, *****p ≤* 0.0001, ns, non significant. CK, cytokeratin; DAPI, 4′,6-diamidino-2- phenylindole; PD1, programmed cell death protein 1.

Supplementary Figure 4 Representative density heat maps of pan macrophages, monocytes, PMNs, CD4^+^ T cells, CD8^+^ T cells, T_regs_, and NK cells infiltrating a chordoma specimen. A large proportion of myeloid cells, T cells, and NK cells are concentrated along the superior and left tumor borders. Many myeloid cells also infiltrate its center. PMNs, polymorphonuclear cells; NK cells, natural killer cells.

Supplementary Figure 5 Chordoma myeloid, T cell, and NK cell density does not significantly differ by age. Immune infiltrate differences between chordoma patients ≤35 years old and >35 years old (n=20) were assessed with a Mann Whitney test using a p value threshold of <0.05. ns, non significant.NK cell, natural killer cell.

Supplementary Figure 6 HALO^®^ spatial proximity analyses of immune cells to chordoma cells demonstrate that T cells are nearer than myeloid cells to tumor cells. (A) Sacral chordoma specimen with three categories of T cells, CD4^+^ T cells, CD8^+^ T cells, and T_regs_ shown in blue, yellow, and red respectively. Tumor cells are shown in green. A segment of heavy T cell infiltrate is magnified. (B) Real time proximity analysis between CD4^+^ T cells (green, Opal 520) and chordoma cells (white, Opal 780) with bars measuring distance between these superimposed on the image in white. (C) Proximity line series between T cells and chordoma tumor cells. (D) Proximity analysis histograms of myeloid cells (top row, red) and T cells (bottom row, blue) to chordoma tumor cells within a 100µm radius by progressive segments of 20µm bands (n=56 individual tumors). Histogram y axes have been standardized for the direct comparison of immune cell phenotype frequencies.
