# Supplementary figures and images for: Multi-spectral immunofluorescence evaluation of the myeloid, T cell, and natural killer cell tumor immune microenvironment in chordoma may guide immunotherapeutic strategies
Source: Front Oncol. 2022 Oct 21;12:1012058. doi: 10.3389/fonc.2022.1012058 (PMC9634172; doi:10.3389/fonc.2022.1012058)

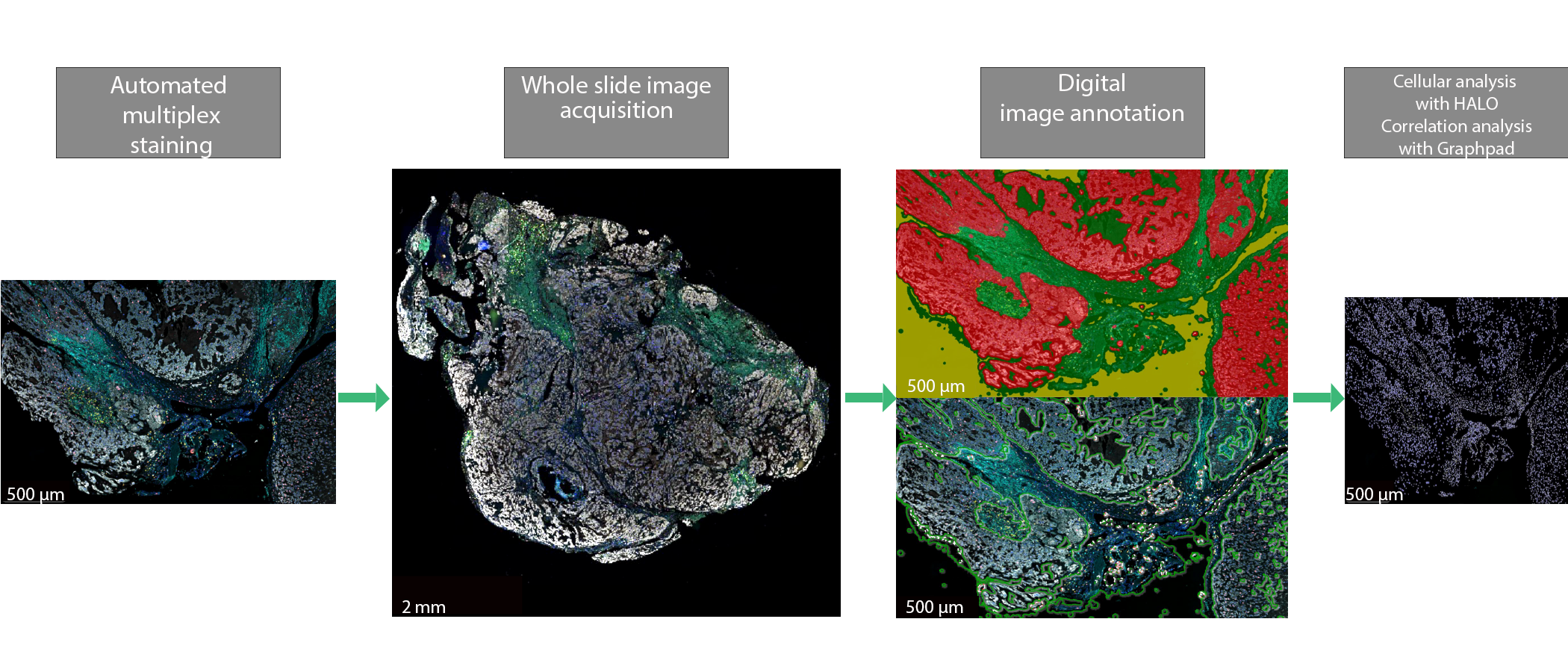

Supplement: Supplementary Figure 1 — Chordoma tissue samples (n=57) were stained using the Leica BOND Rx automated system (PerkinElmer protocol), scanned with VectraPolaris, and analyzed using the HALO® (Indica Labs) v3.3 platform (first panel). For analysis, slides were digitally annotated based on cytokeratin stain (white, Opal 780), which distinguished CK+ chordoma cells from surrounding CK- stroma (third panel). Immune cell quantification, spatial analysis, and clinical comparisons were completed using the HALO® platform and GraphPad Prism version 9.2 (fourth panel). [file Image_1.tif]

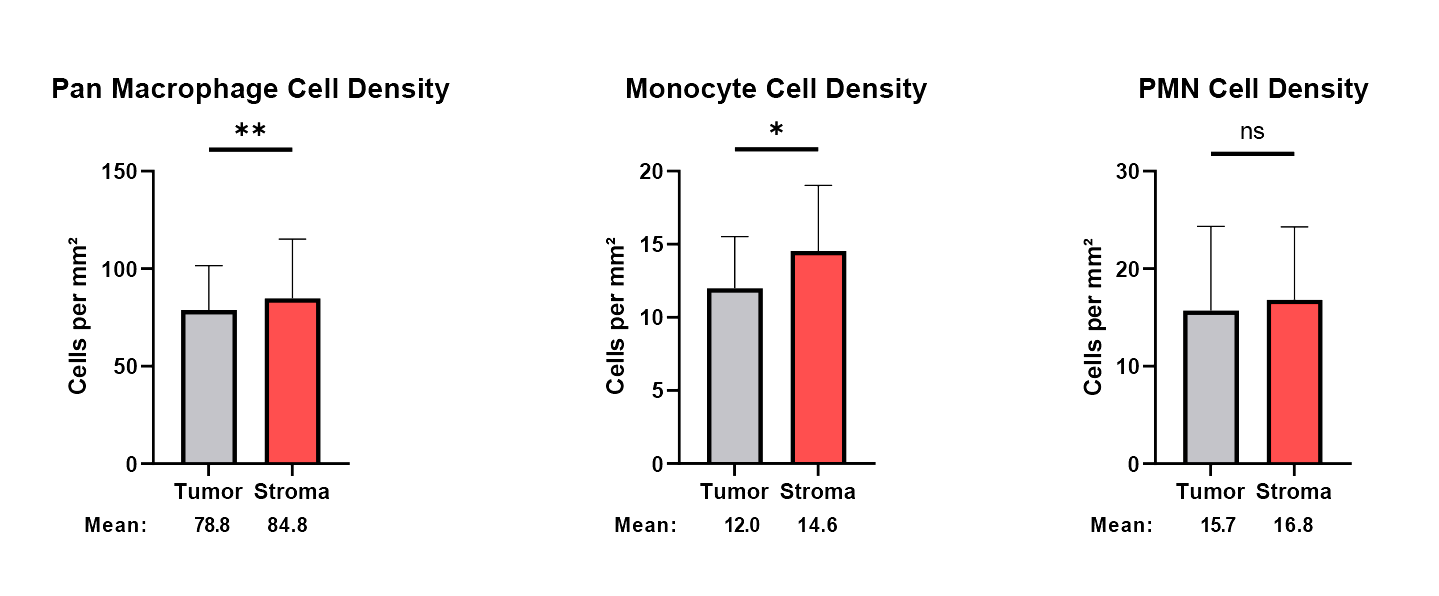

Supplement: Supplementary Figure 2 — Myeloid cells are found within the chordoma tumor immune microenvironment. Quantification of pan macrophages, monocytes, and PMNs per mm2 in chordoma tumor and stroma (n= 56 individual tumors) presented in formatted here as mean ± standard error of the mean. Wilcoxon signed rank tests with p value threshold of <0.05 were used to compare tumor and stroma myeloid cell infiltrate. *p ≤ 0.05, **p ≤ 0.01,ns, non significant. CK, cytokeratin; DAPI, 4′,6-diamidino-2- phenylindole; PMN, polymorphonuclear cells. [file Image_2.tif]

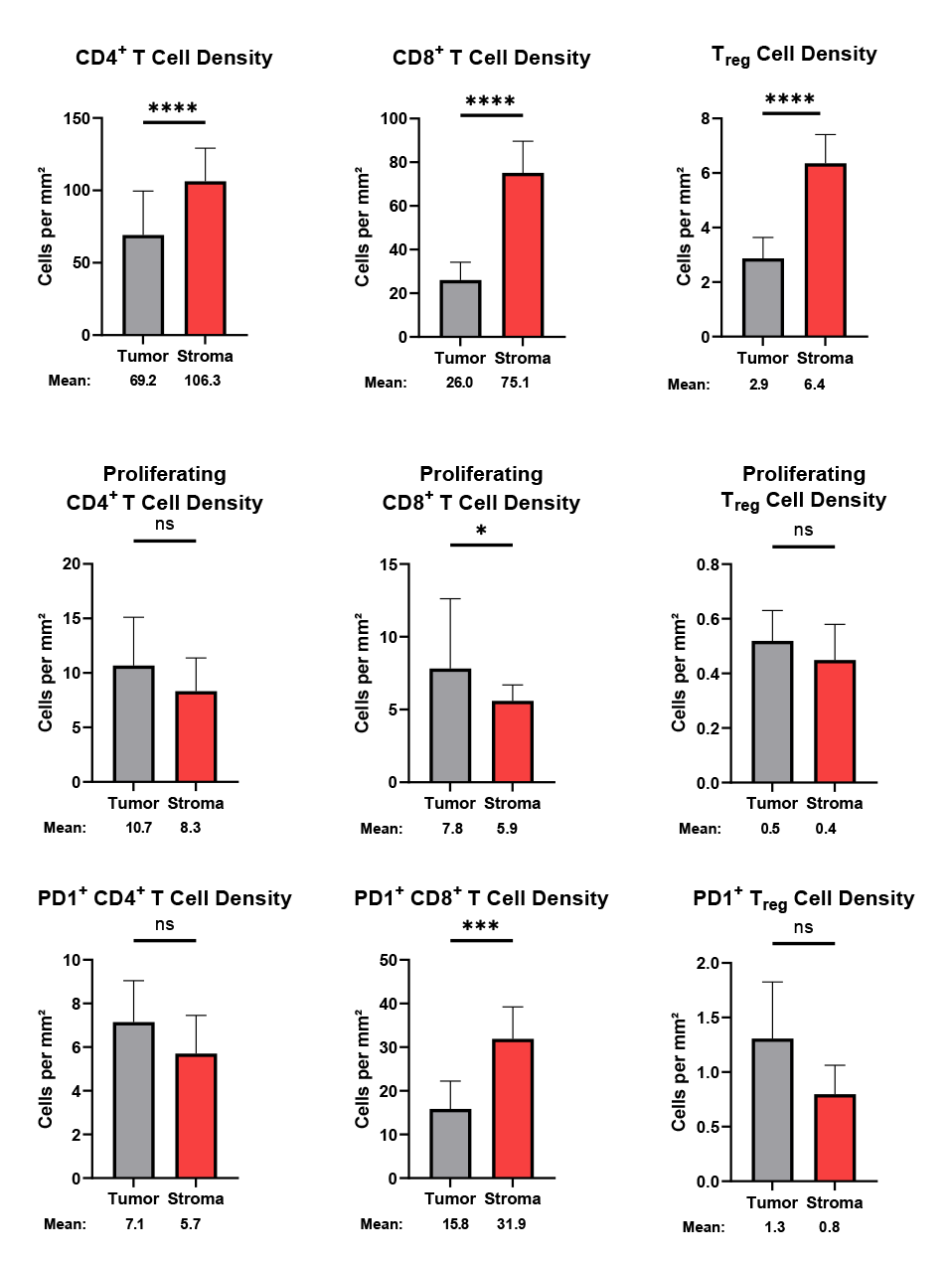

Supplement: Supplementary Figure 3 — T cells are found in abundance within the chordoma tumor immune microenvironment, most often within the stroma. Quantification of CD4+ T cell, CD8+ T cell, and Treg cell density (per mm2), as well as proliferating and PD-1+ subcategories of these are compared between chordoma tumor and stroma (n= 57 individual tumors) presented in formatted here as mean ± standard error of the mean. Wilcoxon signed rank tests with p value threshold of <0.05 were used to compare tumor and stroma T cell infiltrate. *p ≤ 0.05, ***p ≤ 0.001, ****p ≤ 0.0001, ns, non significant. CK, cytokeratin; DAPI, 4′,6-diamidino-2- phenylindole; PD1, programmed cell death protein 1. [file Image_3.tif]

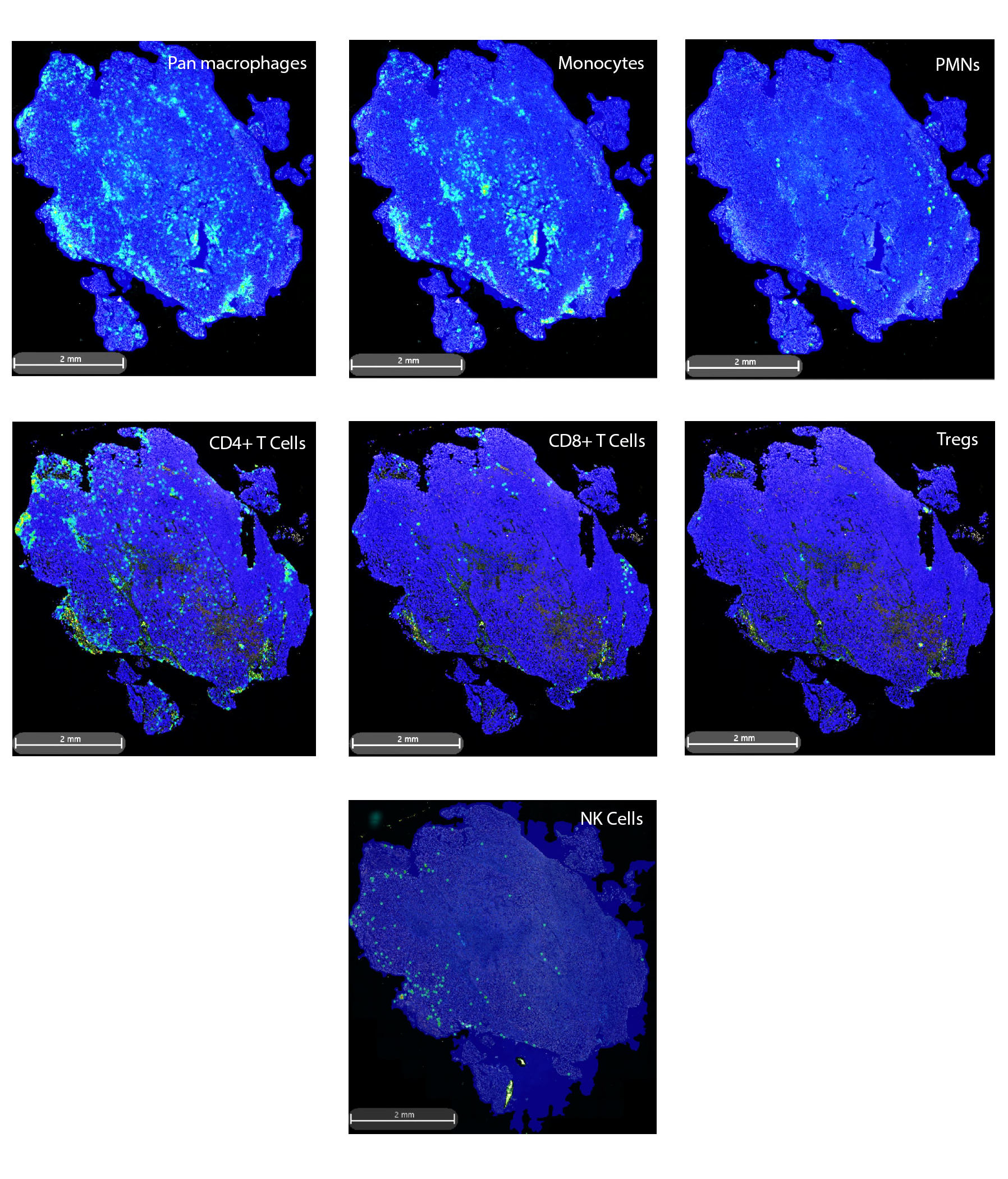

Supplement: Supplementary Figure 4 — Representative density heat maps of pan macrophages, monocytes, PMNs, CD4+ T cells, CD8+ T cells, Tregs, and NK cells infiltrating a chordoma specimen. A large proportion of myeloid cells, T cells, and NK cells are concentrated along the superior and left tumor borders. Many myeloid cells also infiltrate its center. PMNs, polymorphonuclear cells; NK cells, natural killer cells. [file Image_4.tif]

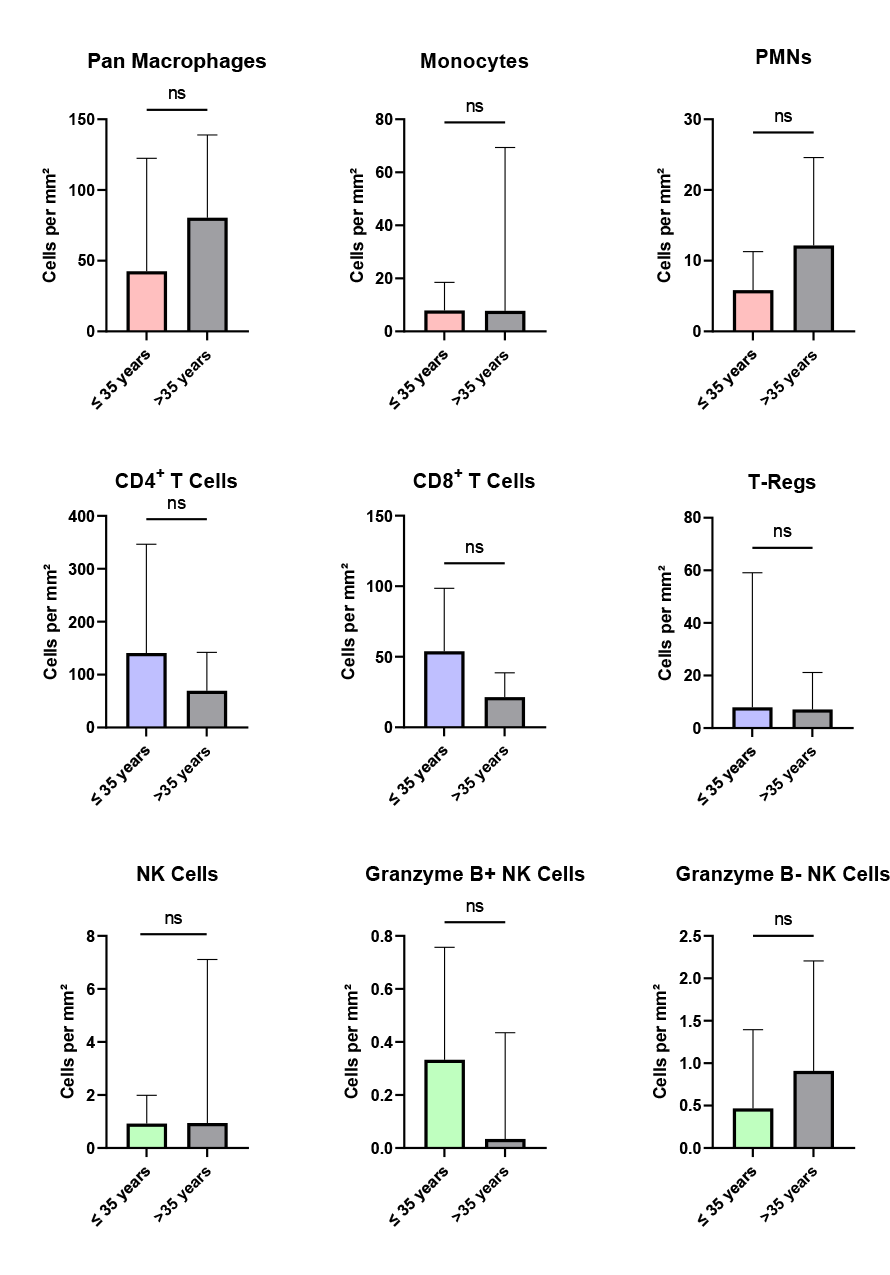

Supplement: Supplementary Figure 5 — Chordoma myeloid, T cell, and NK cell density does not significantly differ by age. Immune infiltrate differences between chordoma patients ≤35 years old and >35 years old (n=20) were assessed with a Mann Whitney test using a p value threshold of <0.05. ns, non significant.NK cell, natural killer cell. [file Image_5.tif]

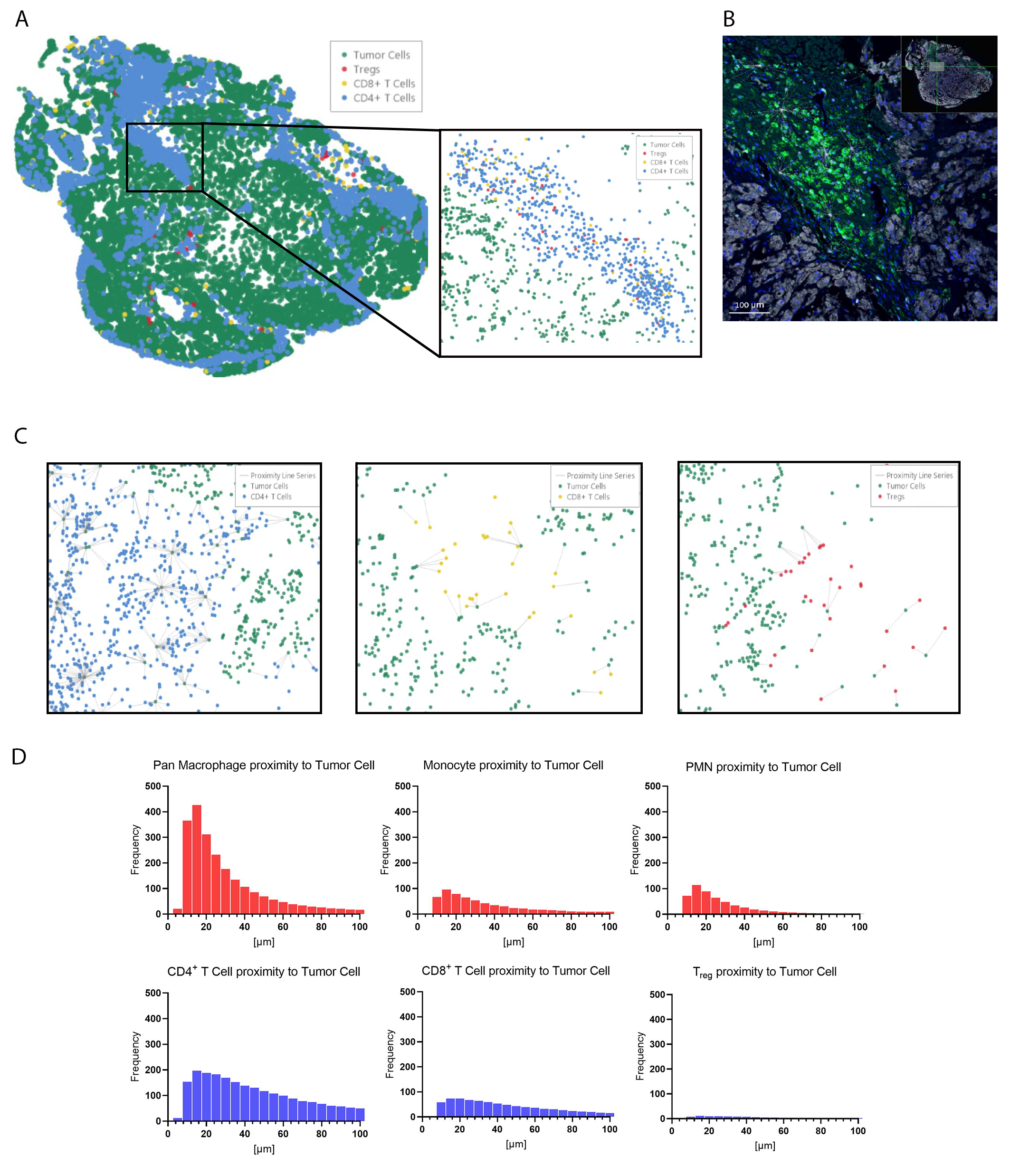

Supplement: Supplementary Figure 6 — HALO® spatial proximity analyses of immune cells to chordoma cells demonstrate that T cells are nearer than myeloid cells to tumor cells. (A) Sacral chordoma specimen with three categories of T cells, CD4+ T cells, CD8+ T cells, and Tregs shown in blue, yellow, and red respectively. Tumor cells are shown in green. A segment of heavy T cell infiltrate is magnified. (B) Real time proximity analysis between CD4+ T cells (green, Opal 520) and chordoma cells (white, Opal 780) with bars measuring distance between these superimposed on the image in white. (C) Proximity line series between T cells and chordoma tumor cells. (D) Proximity analysis histograms of myeloid cells (top row, red) and T cells (bottom row, blue) to chordoma tumor cells within a 100µm radius by progressive segments of 20µm bands (n=56 individual tumors). Histogram y axes have been standardized for the direct comparison of immune cell phenotype frequencies. [file Image_6.tif]
