# Supplementary material for: Multi-spectral immunofluorescence evaluation of the myeloid, T cell, and natural killer cell tumor immune microenvironment in chordoma may guide immunotherapeutic strategies
Source: Front Oncol. 2022 Oct 21;12:1012058. doi: 10.3389/fonc.2022.1012058 (PMC9634172; doi:10.3389/fonc.2022.1012058)
Supplement: Supplementary file 7 [file Table_1.docx]

**Supplementary Table IA – Myeloid Panel**

| Antibody | Vendor/Clone | Catalog # | Dilution 1: | HIER | Secondary Antibody | Opal | Opal Dilution 1: |
| --- | --- | --- | --- | --- | --- | --- | --- |
| CD15 | BD Bioscences [HI98] | #555400 | 1500 |  | Mouse impress | 620 | 150 |
| CD68 | Invitrogen [KP1] | #MA5-13324 | 1000 | ER1/20 min | Akoya Opal Polymer (Akoya Biosciences, #ARH1001EA) | 520 | 150 |
| CD11b | Abcam [EPR1344] | #ab133357 | 5000 | ER1/20 min | Akoya Opal Polymer (Akoya Biosciences, #ARH1001EA) | 570 | 150 |
| CD14 | Abcam [EPR3653] | #ab133335 | 1000 | ER2/20 min | Akoya Opal Polymer (Akoya Biosciences, #ARH1001EA) | 480 | 150 |
| HLA-DR | Abcam [TAL 1B5] | #ab20181 | 1200 | ER1/20 min | Akoya Opal Polymer (Akoya Biosciences, #ARH1001EA) | 690 | 150 |
| Cytokeratin | Santa Cruz [AE1/AE3] | #sc-81714 | 400 | ER2/20 min | Akoya Opal Polymer (Akoya Biosciences, #ARH1001EA) | 780 | 1:50 Opal 780  1:100 TSA-DIG |

**Supplementary Table IB – T Cell Panel**

| Antibody | Vendor/Clone | Catalog # | Dilution 1: | HIER | Secondary Antibody | Opal | Opal Dilution 1: |
| --- | --- | --- | --- | --- | --- | --- | --- |
| CD4 | Abcam [EPR6855] | #ab133616 | 1000 | ER1/20 min | MACH2 (biocare medical, #RHRP520) | 520 | 150 |
| CD8 | Abcam [EPR10640(2) | #ab215041 | 2000 | ER2/20 min | Akoya Opal Polymer (Akoya Biosciences, #ARH1001EA) | 570 | 150 |
| FOXP3 | Invitrogen [SP97] | #MA5-16365 | 200 | ER2/20 min | MACH2 (biocare medical, #RHRP520) | 480 | 150 |
| PD1 | Abcam [EPR4877(2) | #ab137132 | 750 | ER2/20 min | Akoya Opal Polymer (Akoya Biosciences, #ARH1001EA) | 620 | 150 |
| Ki67 | Ventana [30-9] | #790-4286 | 1 | ER1/20 min | MACH2 (biocare medical, #RHRP520) | 690 | 150 |
| Cytokeratin | Santa Cruz [AE1/AE3] | #sc-81714 | 400 | ER2/20 min | Akoya Opal Polymer (Akoya Biosciences, #ARH1001EA) | 780 | 1:50 Opal 780  1:100 TSA-DIG |

**Supplementary Table IC – NK Panel**

| Antibody | Vendor/Clone | Catalog # | Dilution 1: | HIER | Secondary Antibody | Opal | Opal Dilution 1: |
| --- | --- | --- | --- | --- | --- | --- | --- |
| CD3 | Abcam [SP7] | #ab16669 | 300 | ER1/20 min | MACH2 (biocare medical, #RHRP520) | 480 | 150 |
| CD56(NCAM1) | Sigma Aldrich [MRQ-42] | #156R-94 | 250 | ER1/20 min | Akoya Opal Polymer (Akoya Biosciences, #ARH1001EA) | 690 | 150 |
| CD16 | Abcam [SP175] | #ab183354 | 150 | ER2/20 min | Akoya Opal Polymer (Akoya Biosciences, #ARH1001EA) | 620 | 150 |
| Granzyme B | Abcam [EPR8260] | #ab134933 | 200 | ER1/20 min | Akoya Opal Polymer (Akoya Biosciences, #ARH1001EA) | 520 | 150 |
| Cytokeratin | Santa Cruz [AE1/AE3] | #sc-81714 | 400 | ER2/20 min | Akoya Opal Polymer (Akoya Biosciences, #ARH1001EA) | 780 | 1:50 Opal 780  1:100 TSA-DIG |
